# Origin of magnetic properties in carbon implanted ZnO nanowires

**DOI:** 10.1038/s41598-018-25948-x

**Published:** 2018-05-17

**Authors:** Y. F. Wang, Y. C. Shao, S. H. Hsieh, Y. K. Chang, P. H. Yeh, H. C. Hsueh, J. W. Chiou, H. T. Wang, S. C. Ray, H. M. Tsai, C. W. Pao, C. H. Chen, H. J. Lin, J. F. Lee, C. T. Wu, J. J. Wu, Y. M. Chang, K. Asokan, K. H. Chae, T. Ohigashi, Y. Takagi, T. Yokoyama, N. Kosugi, W. F. Pong

**Affiliations:** 1 Department of Physics, Tamkang University, Tamsui, 251 Taiwan; 2 Department of Applied Physics, National University of Kaohsiung, Kaohsiung, 811 Taiwan; 3 Department of Physics, National Tsinghua University, Hsinchu, 300 Taiwan; 4 Department of Physics, University of South Africa, Johannesburg, South Africa; 5 National Synchrotron Radiation Research Center, Hsinchu, 300 Taiwan; 6 Department of Chemical Engineering, National Cheng Kung University, Tainan, 701 Taiwan; 7 Center for Condensed Matter Sciences, National Taiwan University, Taipei, 106 Taiwan; 8 Inter-University Accelerator Center, Aruna Asaf Ali Marg, New Delhi, 110 067 India; 9 Korea Institute of Science and Technology, Seoul, 02792 Republic of Korea; 10 Institute for Molecular Science, Okazaki, 444-8585 Japan

## Abstract

Various synchrotron radiation-based spectroscopic and microscopic techniques are used to elucidate the room-temperature ferromagnetism of carbon-doped ZnO-nanowires (ZnO-C:NW) via a mild C^+^ ion implantation method. The photoluminescence and magnetic hysteresis loops reveal that the implantation of C reduces the number of intrinsic surface defects and increases the saturated magnetization of ZnO-NW. The interstitial implanted C ions constitute the majority of defects in ZnO-C:NW as confirmed by the X-ray absorption spectroscopic studies. The X-ray magnetic circular dichroism spectra of O and C *K*-edge respectively indicate there is a reduction in the number of unpaired/dangling O 2*p* bonds in the surface region of ZnO-C:NW and the C 2*p*-derived states of the implanted C ions strongly affect the net spin polarization in the surface and bulk regions of ZnO-C:NW. Furthermore, these findings corroborate well with the first-principles calculations of C-implanted ZnO in surface and bulk regions, which highlight the stability of implanted C for the suppression and enhancement of the ferromagnetism of the ZnO-C:NW in the surface region and bulk phase, respectively.

## Introduction

Defect-induced room-temperature ferromagnetism (RTFM) in metal oxides has attracted significant attention because of their potential application of such oxides in spintronic devices^1,2^. RTFM in ZnO, TiO_2_, In_2_O_3_ and HfO_2_ and Cu_2_O has been extensively investigated^3–5^. The RTFM-ZnO with its wide band-gap and large exciton binding energy, is one of the most promising materials not only for use in industry but also for scientific interests^6,7^. However, the identification the defect that critically affects the magnetic properties in a ZnO system and the ways such defects facilitate the ferromagnetic coupling are still an open question. Previous investigations have suggested that RTFM may arise from lattice defects [*viz*. O vacancies (V_O_) or Zn interstitials] in pure ZnO thin film, nanoparticles and nanowires^8–10^. Magnetic behavior has been observed in defect-containing ZnO single crystal^11^, in which are critical in inducing magnetic ordering^12,13^. Organic molecule capped ZnO-nanoparticles^14^, nonmagnetic ion-doped ZnO thin films/nanoparticles^15^ and partially oxidized Zn nanowires^16^ also exhibit RTFM behavior. Even the absorption of some organic molecules by ZnO nanoparticles can induce ferromagnetic behavior^17^. The origin of magnetization in ZnO and the role of surface defects^16^, V_O_^6,17,18^, Zn vacancies (V_Zn_)^19,20^, and other defects in the *d*^0^ magnetism mechanism remain controversial. This subject has been recently addressed by Qi *et al*.^21^.

Theoretically, based on density functional theory (DFT) investigations, the origin of magnetism does not involve from Zn 3*d* electrons but does involve the unpaired/dangling 2*p* states of O atoms in the immediate vicinity of V_Zn_, inducing spin polarization at the top of the valence-band (VB)^19^, and the vacancy-induced magnetism preferentially reside on the surface of ZnO^22^. The strong exchange interaction between O 2*p* orbitals and the large effective masses on top of the VB satisfy the Stoner criterion for spontaneous ferromagnetism, which can be made easily by acceptor doping at the O sites in ZnO^23–25^. However, in our earlier report^20^, the *d*^0^ magnetic behavior of ZnO nanocactuses (ZnO-NC) and nanowires (ZnO-NW) was elucidated by using X-ray-based spectroscopic and microscopic measurements, owing to defects in the form of unpaired/dangling O 2*p* states (originally generated by V_Zn_) that induced a significant local spin moment between the nearest-neighboring (NN) O atoms; this finding is supported by the uneven local spin density that was identified from the partial density of states (PDOSs) of O 2*p* states in ZnO using the local density approximation (LDA) and the Hubbard U method. Additionally, according to the theoretical calculations of Yi *et al*.^26^, doping appropriate elements into ZnO can stabilize cation vacancies and form holes, which mediate the ferromagnetism. The C-doped ZnO exhibits intrinsic *n*-type ferromagnetic behavior with a Curie temperature that exceeds room-temperature^15^ and provides a novel way to dope ZnO to form a diluted magnetic semiconductors^27^. The DFT calculation also reveals strong magnetic moments in C-doped ZnO^28^, in which O atoms are substituted with, or replaced by, C atoms stabilizing the ferromagnetism^29^. Calculations from first principles indicate that, in contrast, C atoms that substituted at Zn sites (C_Zn_) in C-doped ZnO, which is thermodynamically more stable than that with C atoms that substituted at O sites (C_O_), form diatomic carbon-complexes with a magnetic moment of 2 μ_B_^30^. A nanosturctural ZnO sample is an excellent system for investigating defects-related phenomena owing to the large surface-to-volume ratio and the presence of various intrinsic defects in the ZnO matrix, such as V_Zn_, V_O_ and others. Implanted C atoms in ZnO nanowires (ZnO-C:NW) provide an ideal opportunity to examine the effect of C implantation on the bonding states and electronic structures of such NWs, based on an in depth understanding of how the implanted C atoms modify magnetic behavior in a ZnO host. However, precisely determining whether implanted C atoms preferentially substitute at either vacancies of V_Zn_ or V_O_ sites to form C_Zn_ or C_O_, respectively, or just form interstitial defects (C_i_) in ZnO matrix is difficult, because defect-variations in the surface and bulk regions that are formed by the implantation of C atoms cause distinct magnetic behaviors in ZnO-C:NW, as mentioned above. To elucidate these issues, spatially-resolved and element-specific synchrotron-based spectroscopic and microscopic techniques are required to provide clear information concerning implanted C atoms and the RTFM of ZnO-C:NW.

In this work, 40 keV ^12^C^+^ ions were implanted into ZnO-NW with a fluence of 1.5 × 10^16^ ions/cm^2^ to form ZnO-C:NW which was examined along with ZnO-NW, respectively. The low implantation energy of 40 keV is selected to avoid any implantation induced defects. X-ray spectroscopic and microscopic techniques were used to probe the effects of defects that were generated by the implantation of C atoms at specific sites and in particular regions, with reference to bonding states, electronic structures and magnetic behaviors of ZnO-NW. Various X-ray techniques that used are the core-level X-ray photoelectron spectroscopy (XPS), X-ray absorption near-edge structure (XANES), scanning photoelectron microscopy (SPEM), scanning transmission X-ray microscopy (STXM), and valence-band photoemission spectroscopy (VB-PES). The main advantage of the use of STXM-XANES herein is its ability to map the chemical states and determine spatially-resolved electronic structures in a selected region and site of interest, and these are typically extracted using image masks that are applied to the STXM data^31,32^. X-ray magnetic circular dichroism (XMCD) measurement and extended X-ray absorption fine structure (EXAFS) spectroscopy were also performed to support the finding that implanted C atoms primarily form C_i_ defects in ZnO-C:NW, greatly suppressing and enhancing magnetization in the surface and bulk regions, respectively, relative to those in ZnO-NW. The results of this investigation provide clear evidence that the various RTFM behaviors between the surface and bulk regions of ZnO-C:NW compared to those of RTFM behaviors of ZnO-NW, depending on not only the population of unpaired/dangling O 2*p*-derived states, but also the population of C 2*p*-derived states of the interstitial C atoms in the ZnO-C:NW.

## Results and Discussion

**Figure 1:**
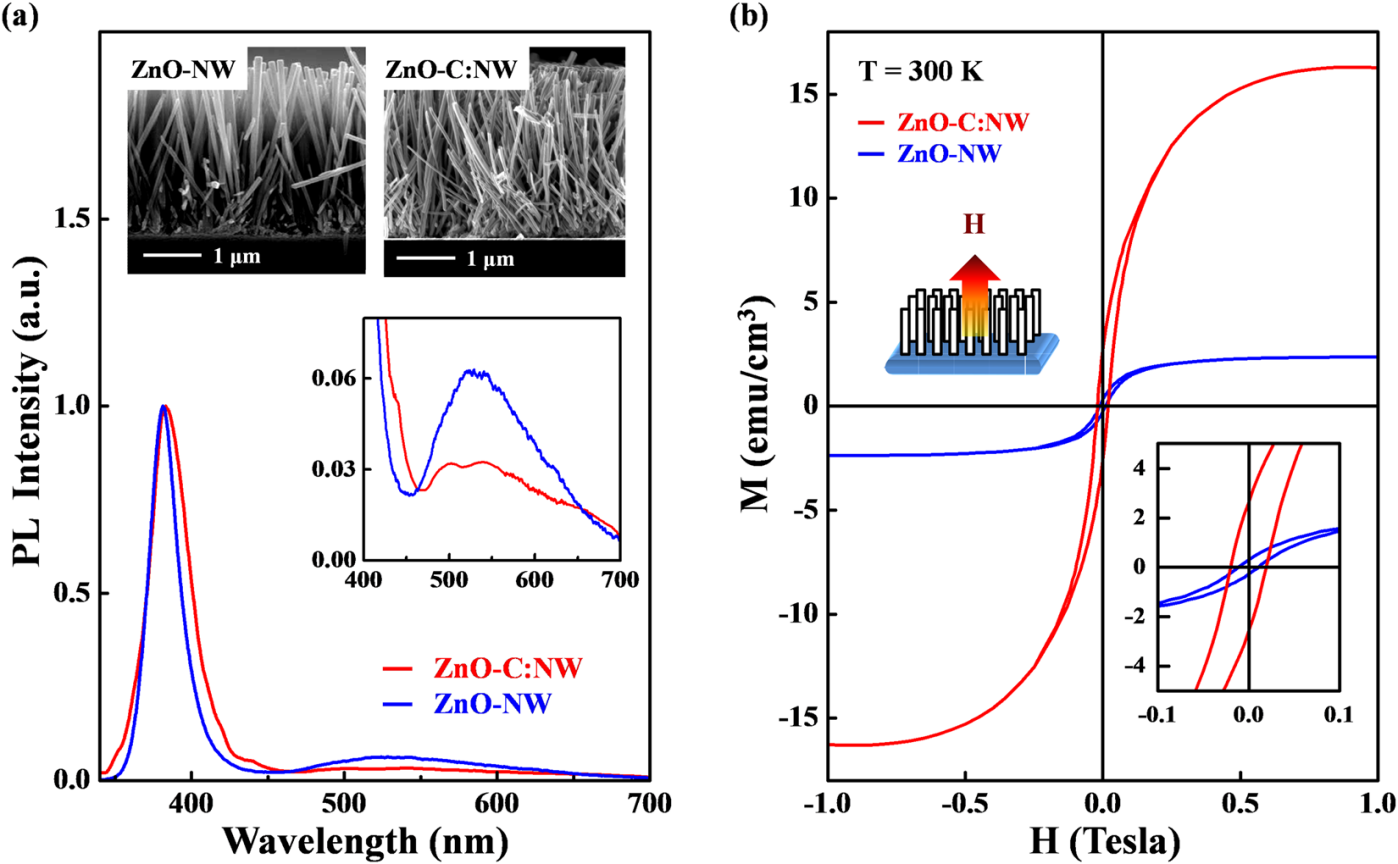
(**a**) PL spectra and cross-sectional SEM images of ZnO-C:NW and ZnO-NW. Inset shows the magnified view of PL range of 400–700 nm. (**b**) Room-temperature M-H curves of ZnO-C:NW and ZnO-NW. Upper and lower insets present magnetic field applied parallel to growth direction (***c***-axis) and magnified M-H loops of ZnO-C:NW and ZnO-NW, respectively, at 300 K.

Figure 1(a) presents the photoluminescence (PL) spectra and cross-sectional scanning electron microscopic (SEM) images of ZnO-C:NW and ZnO-NW. These SEM images reveal that the diameters and lengths of nanostructural ZnO samples are approximately 100 nm and 3 μm^33^, respectively. Clearly, the ZnO-C:NW exhibits a more disordered surface morphology than ZnO-NW. The PL spectra include two features- one with a sharp peak at ~380 nm and another broad feature that is centered at ~540 nm; these are typically attributed to a near-band-edge transition and a broad emission that is caused by surface-related or grain-boundary defects^34–36^, respectively. Although the involvement of various defects such as V_O_^37^, V_Zn_^38^ and O antisites^39^ which can induce green luminescence in ZnO, is still a matter of debate, Janotti *et al*.^38^ reported that, based on DFT and LDA calculations, the low formation energy of V_Zn_ is the major source of green luminescence in ZnO. Obviously, the near-band-edge emission of ZnO-C:NW is shifted to higher wavelengths, owing to the formation of atomic dislocations and/or structural disorder by the implantation of C atoms in the ZnO matrix^40–43^. The inset in Fig. 1(a) shows the magnified view of the broad feature associated with surface-related defects transition within the range 400–700 nm. Interestingly, the intensity of this feature in ZnO-C:NW is significantly lower than that of ZnO-NW. Since the emission from surface defects, centered at ~540 nm is primarily associated with V_Zn_ in ZnO^20,44^, the reduction of intensity of the broad emission feature in the PL spectrum, as displayed in Fig. 1(a), may be caused by the bonding of implanted C atoms to the neighboring unpaired/dangling O 2*p* states of O atoms (surface-related defects), forming C-O bonds by either the substitution of C atoms at the Zn-vacancies or interstitial defects of C_i_. The population of surface-related defects is therefore reduced (and the number of unpaired/dangling O 2*p* bonds or V_Zn_ is likely reduced) in ZnO-C:NW^45^. Figure 1(b) plots the room-temperature magnetic-hysteresis (M-H) curves of both ZnO-C:NW and ZnO-NW. The magnetic field was applied parallel to the direction of growth of the NW, along the ***c-***axis, as shown in the upper inset in the figure. Figure 1(b) reveals that the magnetization of ZnO-C:NW is significantly greater than that of ZnO-NW. The lower inset in Fig. 1(b) clearly reveals the enhanced magnetization in ZnO-C:NW, for which saturation magnetization (M_s_) and coercivity of ZnO-C:NW (ZnO-NW) are ~2.7 emu cm^−3^ (0.3 emu cm^−3^) and ~200 Oe (100 Oe), respectively. The M_s_ for ZnO-C:NW is about ten times that of ZnO-NW, and this effect is attributable to the implanted C atoms in the ZnO-NW matrix. The above results indicate the surprising reduction of intensity of the broad PL emission primarily caused by surface-related defects and an enhancement of magnetization, based on M-H measurements of bulk sensitivity of the ZnO-C:NW relative to ZnO-NW. All these findings suggest that the difference between the effects of implanted C atoms in the surface and bulk regions are responsible for the distinctive PL and magnetic behaviors in the ZnO-C:NW relative to those of ZnO-NW.

Figure S1 in Supplementary Information shows Resonance Rutherford Backscattering Spectrometry (RRBS)^46,47^ spectra that were recorded at an energy of ~4.27 MeV (α-particles), to obtain the elemental ratio depth profiles in ZnO-C:NW and ZnO-NW for reference, further establishing the distribution of implanted C atoms in the surface and bulk regions of ZnO nanostructures. Generally, as presented in Fig. S1(b), the C-depth profile reveals the atomic ratio of C atoms in the 0–400 nm region and most of C atoms are concentrated at the depth of ~200 nm below the surface, indicating that the implanted C atoms are generally in the bulk region in ZnO-C:NW. Based on the RRBS measurement shown in the Fig. S1(b), the Zn- and O-depth profiles do not reveal any significant differences in the atomic distributions of Zn and O in ZnO-C:NW and ZnO-NW, indicating that C atoms that were implanted in ZnO-NW did not significantly change the atomic structure of the ZnO matrix.

**Figure 2:**
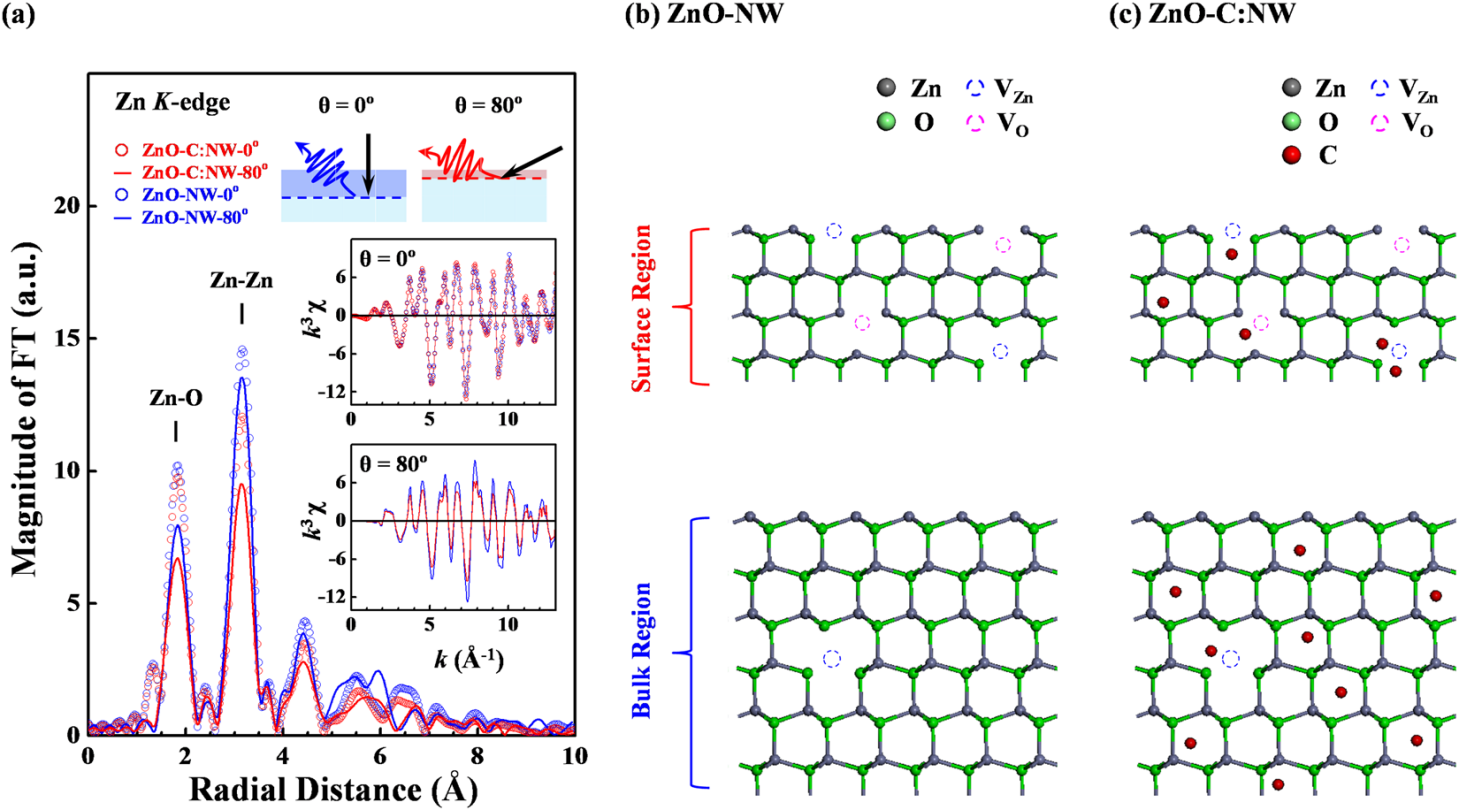
(**a**) Fourier-transformed *k*^3^χ data of Zn *K*-edge EXAFS measurements of ZnO-C:NW and ZnO-NW from *k* = 3.0 to 13.0 Å. Upper insets show angles θ between surface normal and direction of incident X-ray that are selected to obtain local atomic structure in surface and bulk regions. Lower insets display corresponding *k*^3^χ data of Zn *K*-edge EXAFS oscillations. (**b**) Two-dimensional atomic structure of ZnO-NW and **(c)** ZnO-C:NW.

Figure 2(a) displays the Fourier-transformed (FT) spectra of the Zn *K*-edge EXAFS of ZnO-C:NW and ZnO-NW, and their corresponding *k*^3^χ data (lower insets) that were obtained in fluorescence yield mode. As shown in the upper insets in Fig. 2(a), the angles θ between the surface normal and the direction of incident X-ray are selected to obtain the local atomic structure in particular regions, with θ = 80^0^ and 0^0^, which are typically used for probing surface and bulk regions (upper insets), respectively. The Zn *K*-edge FT spectra include two main features at ~1.8 and 3.1 Å, corresponding to the NN Zn-O and Zn-Zn bond distances (without phase correction) in ZnO nanostructures^48,49^. The general line-shape and radial distribution of the FT spectra of the surface and bulk regions of ZnO-C:NW and ZnO-NW are very similar to each other, with no clear broadened or split features associated with the NN Zn-O and Zn-Zn bond distances in the FT spectra of ZnO-C:NW, relative to those in the FT spectra of ZnO-NW. This finding demonstrates that the implanted C atoms in the ZnO-NW matrix do not substitute the vacancies at V_O_ or V_Zn_ sites to form Zn-C bonds in either the first or second shells around Zn atoms. Notably, the atomic radii of C, O and Zn atoms are ~0.91, 0.63 and 1.53 Å^50^, so if C atoms replace the vacancies at V_O_ or V_Zn_ sites to form Zn-C bonds, whose length differs considerably from those of Zn-O or Zn-Zn, broadened or split features will be observed in the Zn *K*-edge FT spectra. Figures S2(a,b) in Supplementary Information present the phase and phase derivative analysis^51–53^ to verify the fact that the implanted C atoms do not substitute the vacancies at either V_O_ or V_Zn_ sites, interstitial C atoms forming Zn-C bonds in first and second nearest neighbor Zn atoms in the ZnO matrix. Phase derivative analysis has been performed extensively on distorted structures^52^, because it provides accurate information about atoms at various sites with slightly different bond distances, yielding a *beating point* (**k**_**b**_) in EXAFS oscillations. Accordingly, if implanted C atoms actually replaced the vacancies at either V_O_ or V_Zn_ sites to form Zn-C bonds in the first or second shells around Zn atoms, then **k**_**b**_ could be observed and extracted from the inverse FT spectra of NN Zn-O and Zn-Zn which are displayed in Fig. S2(a). However, the inverse FT spectra and phase derived analysis of first-shell Zn-O and second-shell Zn-Zn bond lengths in the surface and bulk regions of ZnO-C:NW and ZnO-NW, as presented in Figs. S2(a,b), yield almost identical extracted phase functions Ψ(*k*) and phase derivative functions dΨ/d*k*, which do not yield the **k**_**b**_ in EXAFS oscillations of ZnO-C:NW, eliminate the possibility that implanted C atoms substitute either V_O_ or V_Zn_ sites to form Zn-C bonds at either first or second shells around Zn atoms in ZnO-C:NW. This finding evidently supports the formation of C_i_ in ZnO-C:NW upon the implantation of C atoms. Additionally, comparing the intensities of the FT spectra of ZnO-C:NW and ZnO-NW, presented in Fig. 2(a), clearly reveals the large structural disorder or high Debye-Waller (DW) factors around Zn atoms in ZnO-C:NW, as evidenced by the lower main feature intensities in the corresponding FT spectra than in those of ZnO-NW, because the implanted C atoms formed interstitial defects; induced large structural disorder, or increased DW factors. This result is consistent with the slight shift in the wavelength of emission and the broad near-band-transition in the PL spectrum of ZnO-C:NW, as stated above in relation to Fig. 1(a). The intensities of the FT spectra of the surface and bulk regions of ZnO-C:NW and ZnO-NW reveal that intensities of the first two main features of the FT spectra of the surface region (θ = 80^0^) are much lower than those of the bulk region, primarily because the former contains more defects, causing the higher structural disorder or DW factors than in the bulk region (θ = 0^0^). Specifically, the maximum intensity of the NN Zn-O feature in the FT spectrum of the bulk region of ZnO-C:NW is slightly lower (by ~4%) than that of ZnO-NW; in contrast, the maximum intensity of the NN Zn-O feature in the FT spectrum of surface region of ZnO-C:NW is much lower (by ~16%) than that of ZnO-NW. This phenomenon follows from the fact that the NN Zn-O shell around Zn atoms in the bulk region of ZnO-C:NW has fewer defects (the bulk ZnO matrix is almost perfect), so the DW factors are smaller and closer to those of ZnO-NW. Moreover, the maximum intensity of the NN Zn-Zn shell in the FT spectra of the bulk and surface regions of ZnO-C:NW are ~18% and 30% lower than those of ZnO-NW, respectively, indicating that the increase in DW factors by the implantation of C atoms at the NN Zn-Zn shell in the surface region exceeds that in the bulk region; a similar result is obtained with respect to behavior associated with the NN Zn-O shell around the Zn atoms in ZnO-C:NW and ZnO-NW. Based on the Zn *K*-edge EXAFS data, the surface region typically exhibits greater structural disorder or larger DW factors than the bulk region in both ZnO-C:NW and ZnO-NW. Implanted C atoms in the surface of region of ZnO-C:NW which induced more structural distortion and larger DW factors than those in the bulk region, although many of the implanted C atoms are distributed in the bulk region, according to RRBS measurements. Based on the EXAFS results, Fig. 2(b) schematically depicts a two-dimensional atomic structure of ZnO-NW with implanted C atoms, which primarily form interstitial defects of C_i_ in the resulting ZnO-C:NW. Various intrinsic defects of V_Zn_ and V_O_ in the surface and bulk regions of ZnO-NW [Fig. 2(b)] and ZnO-C:NW [Fig. 2(c)] are also presented.

**Figure 3:**
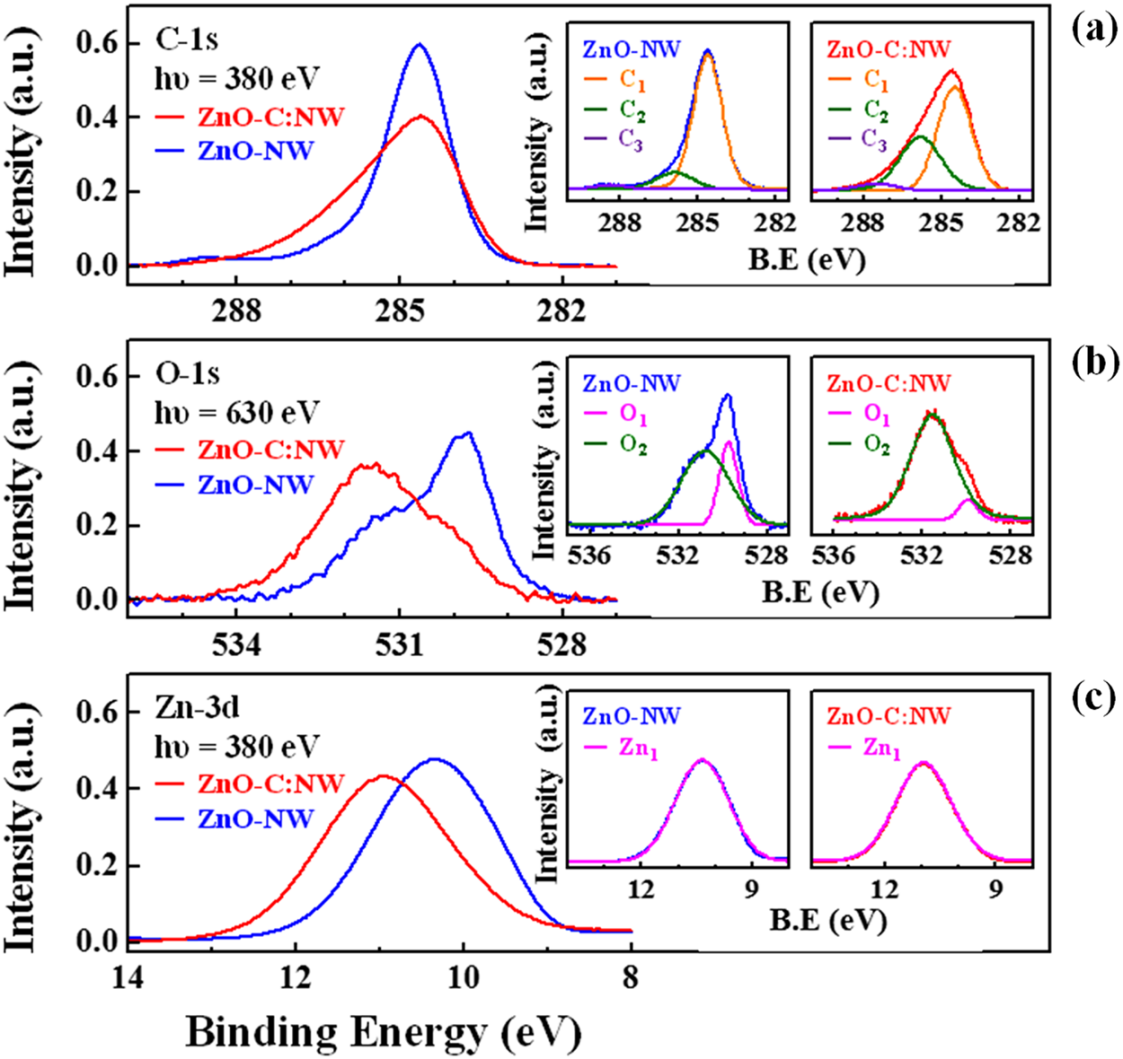
Core-level XPS spectra of (**a**) C 1 *s*, (**b**) O 1 *s* and (**c**) Zn 3*d* states with fitted bonding states of ZnO-C:NW and ZnO-NW.

Figure 3 presents the core-level XPS spectra of C, O 1 *s* and Zn 3*d* states along with their fitted bonding states of ZnO-C:NW and ZnO-NW. Owing to C-contamination at the surface, C 1 *s* XPS analysis based on the survey scan of ZnO-NW reveals the feature at ~284.5 eV, as shown in Fig. 3(a)^54^. Furthermore, the line-shape of the main feature of the C 1 *s* state in the XPS spectrum clearly changes upon implantation, becoming broader, implying C-related chemical states are induced in the surface region by the implantation of C atoms in ZnO-C:NW. The insets in Fig. 3(a) present the fitted C 1 *s* XPS spectra of ZnO-C:NW (ZnO-NW), including three features, which are attributed to C_1_ ~284.6 (284.5) eV, C_2_~285.9 (285.8) eV and C_3_~288.4 (287.5) eV, which are typically associated with C=C, C-O and C=O bonds^55,56^, respectively. Notably, the C 1 *s* XPS spectra of ZnO-C:NW show that the intensity of feature C_2_ (C-O bond) is significantly greater than that of ZnO-NW, suggesting the modification of chemical states upon C implantation in the surface region. The main features of the O 1 *s* and Zn 3*d* states in the XPS spectra of ZnO-C:NW are shifted to higher binding energies than those of ZnO-NW, as presented in Fig. 3(b,c). The insets in Fig. 3(b) display the fitted O 1 *s* XPS spectra of ZnO-C:NW (ZnO-NW) with two features, O_1_ ~529.9 (529.8) eV and O_2_ ~531.5 (530.8) eV, which are typically attributed to O-Zn bonding states and adsorbed O species or hydroxyl groups respectively^55^. The intensity of the O_2_ feature in the O 1 *s* XPS spectrum is increased and is associated with the C_2_ feature in the C 1 *s* XPS spectrum of ZnO-C:NW, clearly supporting the fact that C implantation generates more C-O related bonds in the surface region. This enhancement of features C_2_ and O_2_ in the XPS spectra of ZnO-C:NW is caused by the formation of interstitial defects C_i_ by implanted C atoms, as revealed by the results of the analysis of the Zn *K*-edge EXAFS above. Therefore, the formation of C-O bonds is favored, and the number of unpaired/dangling bonds at O sites is reduced, reducing the surface defects transitions of broad PL feature in ZnO-C:NW, as displayed in Fig. 1(a). The insets in Fig. 3(c) present the Zn 3*d* binding state in ZnO-C:NW and ZnO-NW; the Zn 3*d* binding state in ZnO-C:NW is shifted to a ~1 eV higher binding energy and the width of the XPS feature remain almost unchanged from that of ZnO-NW, suggesting the presence of interstitial C atom around Zn atoms which are in Zn-O bonds, causing the electron of Zn to be shared with the nearby C atoms, thus increasing energy of the Zn 3*d* state. Meanwhile, the Zn 3*d* XPS spectrum of ZnO-C:NW show similar features of ZnO-NW, indicating the absence of the formation of any considerable number of Zn-C chemical bonds by the implantation of C atoms in ZnO-C:NW, further eliminating the possibility that implanted C atoms are substituted at V_O_ or V_Zn_ sites to form Zn-C bonds, which is consistent with the EXAFS results discussed above. Notably, the surface-sensitive XPS measurements (Fig. 3) typically reflect bonding states at/near the surface, and the analysis results herein are consistent with the broad PL emission, but these differ from the bulk-sensitivity experimental M-H curve measurements enhanced magnetization in ZnO-C:NW, as presented in Fig. 1(b).

**Figure 4:**
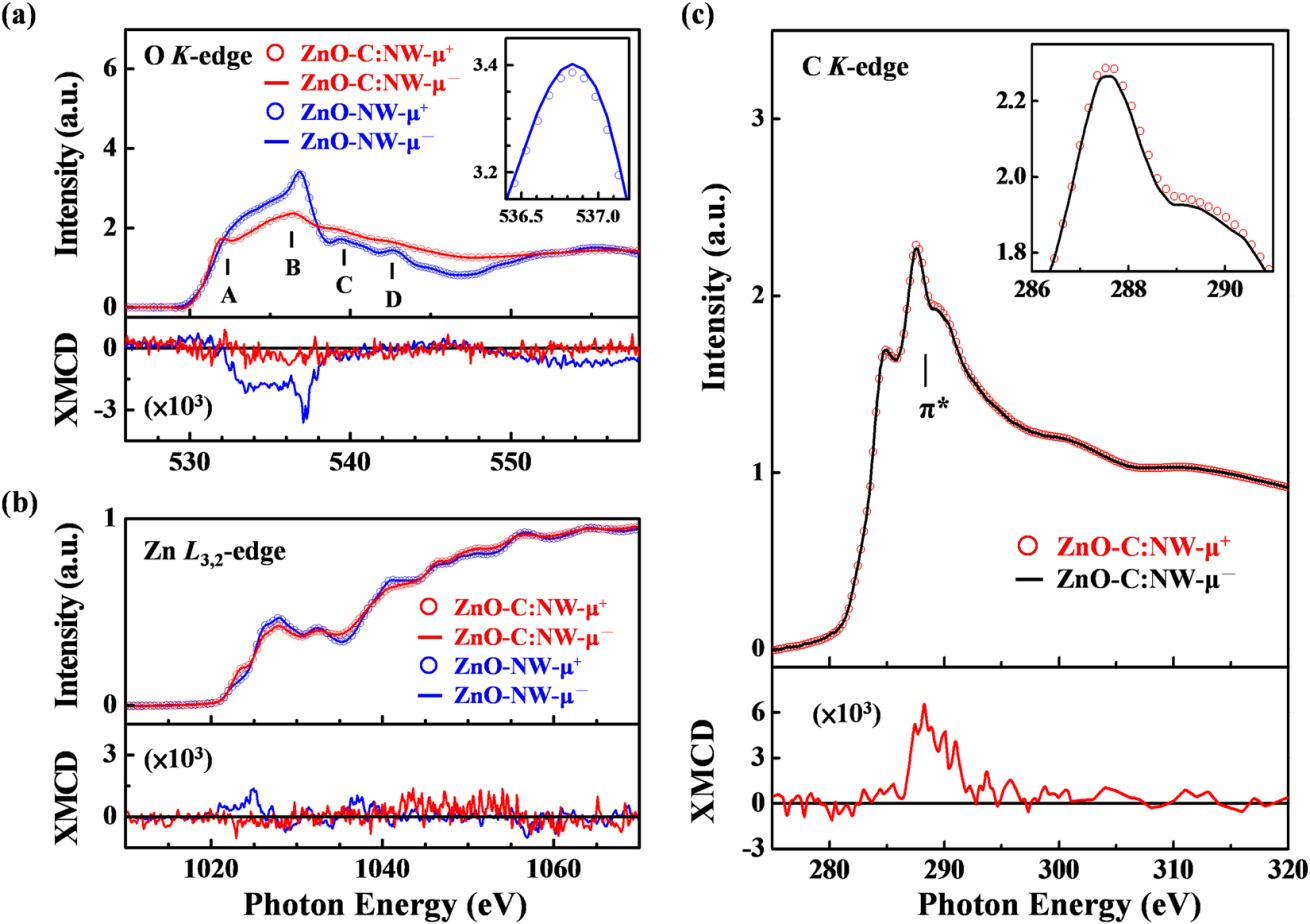
(**a)** Normalized O *K*-edge and (**b)** Zn *L*_3,2_-edge XANES spectra with photon helicity of incident X-rays parallel (μ_+_) and anti-parallel (μ_−_) to direction of magnetization for ZnO-C:NW and ZnO-NW. Upper inset in Fig. 4(a) displays magnified O *K*-edge XANES spectra; lower panels in Fig. 4(a,b) display O *K*-edge and Zn *L*_3,2_-edge XMCD spectra of ZnO-C:NW and ZnO-NW. (**c**) C *K*-edge XANES spectra of ZnO-C:NW. Upper inset magnified view C *K*-edge near-edge spectra of ZnO-C:NW. Lower panel displays C *K*-edge XMCD spectra of ZnO-C:NW.

Figure 4(a,b) present the O *K*-edge and Zn *L*_3,2_-edge XANES spectra of ZnO-C:NW and ZnO-NW, obtained in surface-sensitive total electron yield (TEY) mode, respectively. The lower panels display the corresponding XMCD spectra obtained with the photon helicity of the incident X-rays parallel (μ^+^) and antiparallel (μ^−^) to the direction of magnetization. XMCD spectrum is defined as the ratio of (μ^+^−μ^−^)/(μ^+^+μ^−^). The general line-shapes and positions of the features in the O *K*-edge XANES and XMCD spectra in Fig. 4(a) are consistent with the features of ZnO nanostructures that were obtained in our earlier study^20^. According to the dipole-transition selection rule, the features within the range 530–545 eV, denoted as **A**–**D** in the O *K*-edge XANES spectra, are attributed to the electron excitation from O 1 *s* states to 2*p*_σ_ (along the bilayer) and 2*p*_π_ (along the ***c***-axis) states, and their intensities are approximately proportional to the density of the unoccupied O 2*p*-derived states^57,58^. Typically, the enhancements of features **A**–**D** represent increases in local DOSs that are caused by the defects and unpaired/dangling O 2*p*-derived states in the surface region of the ZnO nanorods^58^. As displayed in Fig. 3(a), in the O *K*-edge XANES spectra of ZnO-C:NW, that the intensities of features **A**–**D** are drastically lower than those of ZnO-NW. The suppression of intensities of features **A**–**D** in the O *K*-edge XANES spectra clearly show the fewer defects in the surface region of ZnO-C:NW. This is because the implanted C atoms reduce the number of unpaired/dangling O 2*p* bonds as the interstitial defects of C_i_ form C-O bonds in the surface region, as discussed above. It is evident from O *K*-edge XANES that the density of the O 2*p*-derived states is lower in the surface region at/above the conduction-band minimum (*E*_CBM_) or Fermi level (*E*_F_) in ZnO-C:NW than that of ZnO-NW. The VB spectra, obtained by SPEM measurement^59^, also revealed that the DOS at/below the valence-band maximum (*E*_VBM_) or *E*_F_ of ZnO-C:NW is lower than that ZnO-NW. Figure S3 in Supplementary Information presents the VB DOS at/below the *E*_VBM_ or *E*_F_ of ZnO-C:NW and ZnO-NW. Since the defects and unpaired/dangling O 2*p* states dominate the occupied states at/below *E*_VBM_ or *E*_F_ in ZnO-C:NW, the strong reduction of the main feature at/below *E*_VBM_ or *E*_F_ of ZnO-C:NW is caused by the lowering of the density of the O 2*p-*derived states. Combining the local DOSs at/above *E*_CBM_ and below *E*_VBM_ or *E*_F_ obtained respectively from the O *K*-edge XANES and the VB SPEM measurements support the claim that implanted C atoms form interstitial defects of C_i_, so some C atoms become bonded to O atoms, reducing the number of unpaired/dangling O 2*p*-derived states in ZnO-C:NW. This finding is consistent with the results concerning the surface sensitivity of PL and with the XPS measurements above. Additionally, as revealed by the XMCD spectra in the lower inset in Fig. 4(a), the weak but confirmed magnetic moment at O sites, caused the imperfect alignment of spin moments of unpaired/dangling O 2*p*-derived states in the surface region of ZnO-NW^20^. Importantly, the intensity of the O *K*-edge XMCD spectrum of ZnO-C:NW is lower than that of ZnO-NW, revealing no clear spin moment of the O 2*p-*derived states in ZnO-C:NW. This result is highly consistent with the above claim that the *d*^0^ magnetic behavior of ZnO-NW is closely related to the number of unpaired/dangling O 2*p*-derived states. However, the results of surface-sensitive XMCD are clearly inconsistent with the measurements of the bulk-sensitivity of M-H, where the M_s_ value of ZnO-C:NW is much higher than that of ZnO-NW. This difference suggests that the magnetic behavior in ZnO-C:NW is not only determined by the number of unpaired/dangling O 2*p*-derived states in the surface region but also affected by implanted C atoms in bulk region. Therefore, the distinctive magnetic behaviors reflect the reduction in the magnetization of the ZnO-C:NW by the O *K*-edge XMCD spectrum (surface-sensitivity), but the enhancement in its M_s_ value by the bulk-sensitive M-H curves, relative to those of ZnO-NW. As displayed in Fig. 4(b), according to the dipole-transition selection rule, since Zn 3*d* is fully occupied in the Zn *L*_3,2_-edge XANES spectra, which are used to probe of unoccupied Zn *s*- and *d*-derived states, the lowest unoccupied orbital of the Zn ion is Zn 4 *s*, which is followed by Zn 4*pd*. The features in the Zn *L*_3,2_-edge XANES spectra are primarily associated with the transition of Zn 2*p* electrons to Zn 4*d*/*s*-derived states^57,58^. The variation in the line-shapes of the Zn *L*_3,2_-edge XANES of ZnO-C:NW can be caused by the change in the electronic structures upon the formation of Zn-C bonds around Zn sites when C atoms are implanted^60^. However, as revealed by the Zn *L*_3,2_-edge XANES and XMCD spectra of ZnO-C:NW, shown in Fig. 4(b) and its lower panel, the XANES and XMCD spectra of Zn 4*d* states of both ZnO-C:NW and ZnO-NW exhibit no clear difference, excluding the possibility that the Zn-*d* orbital has any role in *d*^0^ magnetism in ZnO-C:NW and ZnO-NW, and possibility that implanted C atoms are substituted at V_O_ or V_Zn_ sites to form Zn-C bonds in the first and second shells around Zn atoms in ZnO-C:NW, as discussed above. Figure 4(c) presents the C *K*-edge XANES spectra of ZnO-C:NW that are also obtained the surface-sensitive TEY mode. The C *K*-edge XANES features in Fig. 4(c) in the regions 284–290 eV and 290–300 eV are known to be associated with the C 1 *s* → 2*p*(π*) and 1 *s* → 2*p*(σ*) transitions, respectively^61,62^. The lower panel also displays the corresponding C *K*-edge XMCD spectrum that was obtained with the photon helicity of the incident X-rays parallel (μ^+^) and antiparallel (μ^−^) to the direction of magnetization. The difference between μ^+^ and μ^−^ intensities was magnified in the upper inset of Fig. 4(c). The lower panel in Fig. 4(c) displays the C *K*-edge XMCD spectra [(μ^+^−μ^−^)/(μ^+^_+_μ^−^)] of ZnO-C:NW, clearly revealing that the C 2*p*-derived states of the C atoms affect the magnetic behavior in ZnO-C:NW. Notably, ZnO-NW yields no detectable C *K*-edge XANES and XMCD spectra, although C 1 *s* XPS analysis of ZnO-NW shows a strong feature at ~284.5 eV owing to C-contamination of the surface. As shown in the lower panel in Fig. 4(c), a magnetic moment that was associated with the C 2*p*-derived states in ZnO-C:NW was also observed. The intensity of the C *K*-edge XMCD features in the range 280–292 eV, typically attributed to C 2*p*(π*)-derived states, but no clear XMCD feature of 2*p*(σ*) states was observed in the region 292–300 eV. The C 2*p*(π*)-derived states are clearly responsible for the magnetism of C sites in ZnO-C:NW. The spectral intensities of the XMCD features of O and C *K*-edge are opposite in Fig. 4(a,c), respectively. This result can be explained by the different projected spin contributions of the O 2*p*- and C 2*p*-derived states in ZnO-C:NW, which cause the magnetic moment of the implanted C atoms to align antiparallel to that of the O atoms, possibly weakening the magnetic moment from O sites in the surface region. This phenomenon may also explain the lower panel in Fig. 4(a) exhibits lower magnetization in the O *K*-edge XMCD spectrum of ZnO-C:NW than in that of ZnO-NW. The *d*^0^ magnetic moments of ZnO-NW at O sites have been demonstrated, based on O *K*-edge XMCD measurements^20^, and they result in the unpaired/dangling O 2*p*-derived states, as addressed above. Notably, The integrated XMCD intensity at the C *K*-edge (region of ~287–292 eV) of ZnO-C:NW, presented in the lower panel of Fig. 4(c), is approximately 2.5 times that of the integrated XMCD intensity at the O *K*-edge (region of ~532–538 eV) in the lower panel of Fig. 4(a), revealing that the C 2*p*-derived states of implanted C atoms may critically affect the net spin polarization in the surface and bulk regions of ZnO-C:NW. In particular, based on the results of the RRBS analysis, the C-depth profile indicates that most implanted C atoms in ZnO-C:NW are within the depth of 0–400 Å. These results provide evidence not only that the magnetic moments in the surface or bulk regions are determined by the population of unpaired/dangling O 2*p*-derived states, but also that these moments are associated with the population of C 2*p*-derived states [C 2*p*(π*) states] of the implanted C atoms particularly in the bulk region, explaining the difference between the magnetic behaviors in the surface and bulk regions of ZnO-C:NW and those in ZnO-NW. Spatially-resolved microscopic and spectroscopic techniques are used to provide more information about the effect of implanted C atoms on the difference between the surface and bulk regions in ZnO-C:NW.

**Figure 5:**
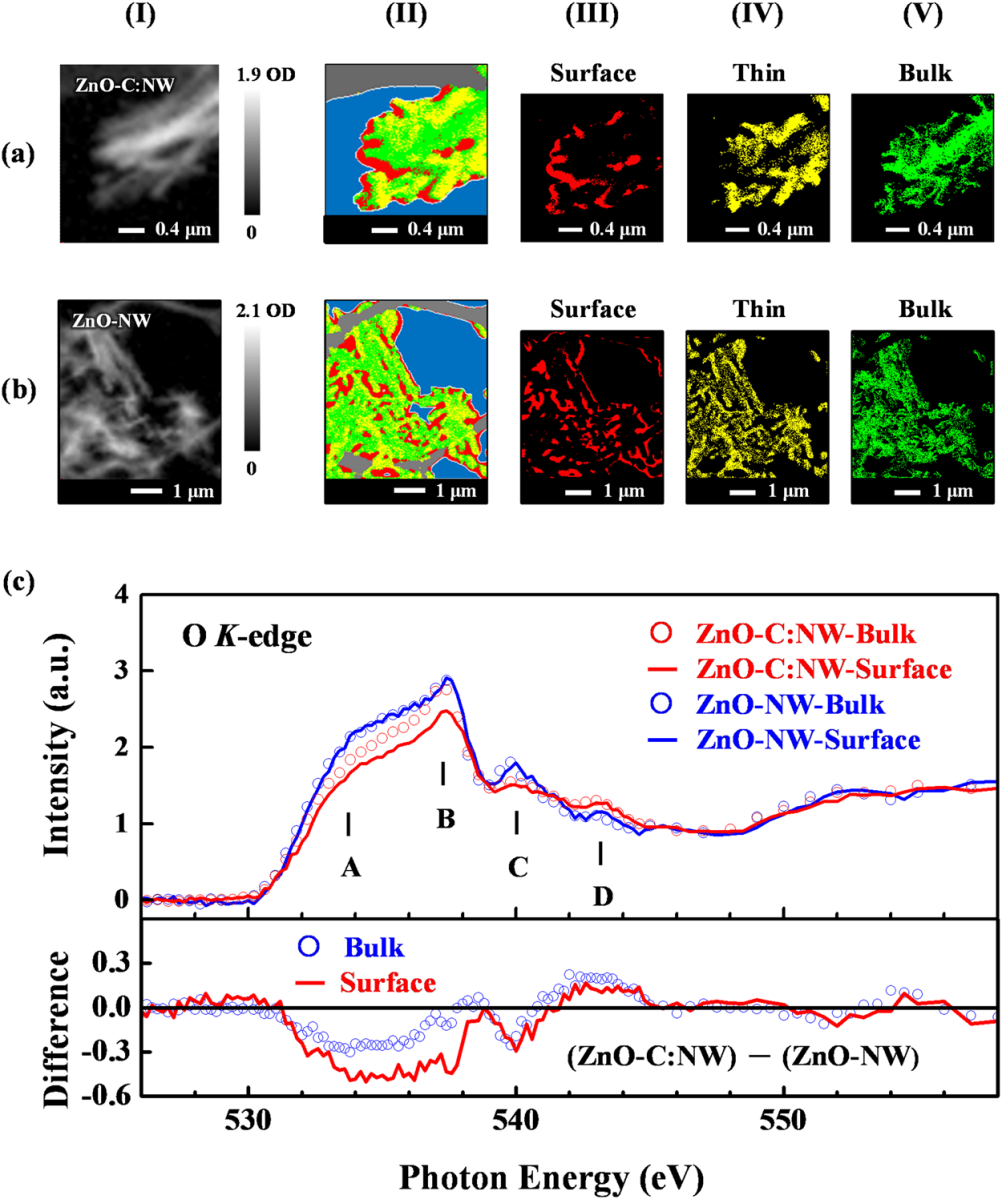
(**a**) Optical density (OD) images (panel I) and (**b**) O *K*-edge STXM stack mapping (panel II) and decomposed STXM mapping (panels III-V) of randomly selected regions of ZnO-C:NW and ZnO-NW. (**c**) O *K*-edge STXM-XANES spectra of surface and bulk regions of ZnO-C:NW and ZnO-NW. Lower panel presents the difference between O *K*-edge STXM-XANES spectra of ZnO-C:NW and ZnO-NW for both surface and bulk regions.

Figure 5(a,b) present the optical density (OD) images (panel I), O *K*-edge STXM stack mapping (panel II) and decomposed STXM mapping (panels III-V) of randomly selected regions of ZnO-C:NW and ZnO-NW. The OD images of ZnO-C:NW [panel I(a)] and ZnO-NW [panel I(b)] show three different regions namely: (a) the bright areas, the thick regions, (b) the dim areas, the thin regions and (c) the grey areas, intermediate thickness. The stack mappings (panel II) are decomposed into red (panel III), yellow (panel IV) and green (panel V) maps, which correspond to the regions that are associated with different thicknesses and spectroscopic variations of the NWs. The different regions of the mappings thin-primarily the surface/edge (red), medium (yellow) and thick-primarily the bulk (green) regions (predominantly the perfect ZnO structure) were generated by principle composition analysis (PCA) for cluster analysis, based on spectroscopic differences. More details of STXM measurements and analysis of the data concerning various nanomaterials can be found elsewhere^20,32,62^. Figure 5(c) presents the O *K*-edge STXM-XANES spectra of the surface and bulk regions of ZnO-C:NW and ZnO-NW. These spectra are the sums of corresponding XANES spectra of the red and green regions of ZnO-C:NW and ZnO-NW, respectively, shown in panels III and V in Fig. 5(a,b). The general line shapes and features in the O *K*-edge STXM-XANES spectra are consistent with the previous nanostructural ZnO study^20^. The O *K*-edge STXM-XANES spectra that are in Fig. 5(c) clearly reveal that the intensities of features **A**–**D** in the surface and bulk regions of ZnO-C:NW are much lower than those of ZnO-NW, as consistent with the O *K*-edge XANES in Fig. 4(a). To compare the variations of O *K*-edge STXM-XANES features in the surface and bulk regions of ZnO-C:NW with those in the surface and bulk regions of ZnO-NW, the lower panel in Fig. 5(c) presents the different between the O *K*-edge STXM-XANES spectra of the surface and bulk regions of ZnO-C:NW and those of ZnO-NW. The difference spectrum for the surface region that includes features **A**–**C** is more negative than that in the bulk region. Since the surface and bulk regions have similar orientations in ZnO-C:NW and (initial) ZnO-NW, the effect of polarization can be ignored and the difference can be attributed to the greater reduction in the number of O 2*p*-derived states in the surface region of ZnO-C:NW by implanted C atoms than in the bulk region. As discussed above, the large suppression of the features in the O *K*-edge STXM-XANES spectra of the surface region of ZnO-C:NW reflected a more reduction of the population of unpaired/dangling O 2*p-*derived states because implanted C atoms favored the formation of C-O bonds in the surface region. Since the population of unpaired/dangling bonds at the O sites in the surface region generally exceeded that in the bulk region, the implanted C atoms had more opportunity to form C-O bonds there, so the number of unpaired/dangling O 2*p* bonds was reduced to a greater extent, thus the intensity of the features in the O *K*-edge STXM-XANES in the surface region was reduced to a greater extent. In contrast, in the bulk region (with an almost perfect ZnO structure), the population of unpaired/dangling O 2*p*-derived states, forming C-O bonds, was lower, so the implanted C atoms primarily formed interstitial defects of C_i_, as presented in Fig. 2(b); therefore, the unpaired/dangling C 2*p*(π*)-derived states of the implanted C atoms in the bulk region, rather than the unpaired/dangling O 2*p*-derived states in the surface region, importantly affects the magnetic behavior of ZnO-C:NW. Clearly, the implanted C atoms dominated the observable reduction in the magnetic moment of the O *K*-edge XMCD in the surface region and the enhancement in the M-H curve (bulk-sensitive) of the ZnO-C:NW, relative to ZnO-NW. The unpaired/dangling O 2*p*-states around Zn-vacancies have been theoretically identified as key contributors to *d*^0^ magnetism in ZnO^20^. However, a finer mechanism is necessary to explain the implanted C-induced differences in the observed magnetic behaviors in the surface and bulk regions of ZnO-C: NW, respectively. First-principles spin-polarized total-energy calculations of wurzite ZnO with point defects using the plane-wave-based VASP package^63^ were performed herein to elucidate the effects of the defects that are formed by implanted C atoms on the electronic structures and magnetic properties of ZnO-C:NW. An efficient generalized-gradient approximation plus Hubbard U (GGA + U) method^64^ is used to quantify the strong Coulomb correlation between the 3*d* electrons of Zn cations and O 2*p* hole states when ZnO is doped with Zn-vacancies. Calculations concerning defects in the bulk region are carried out using a 72-atom 3x3x2 wurtzite supercell [Zn_36_O_36_, as shown in Fig. S4(a)]. One of the stable and commonly observed surfaces of ZnO NW is a nonpolar prism $(11\overline{2}0)$ surface^65^. Therefore, a seven-layer atomic slab (Zn_56_O_56_) with a $[11\overline{2}0]$ surface orientation and a vacuum space of 15 Å [as shown in Fig. S4(b)] is used to simulate defect configurations in the surface region of ZnO-NW. The stability of a specific defect configuration depends strongly on its formation energy, which can be determined by DFT calculations from first-principles without experimental data. The Supplementary Information presents details of the calculations of total energy and formation energy.

**Figure 6:**
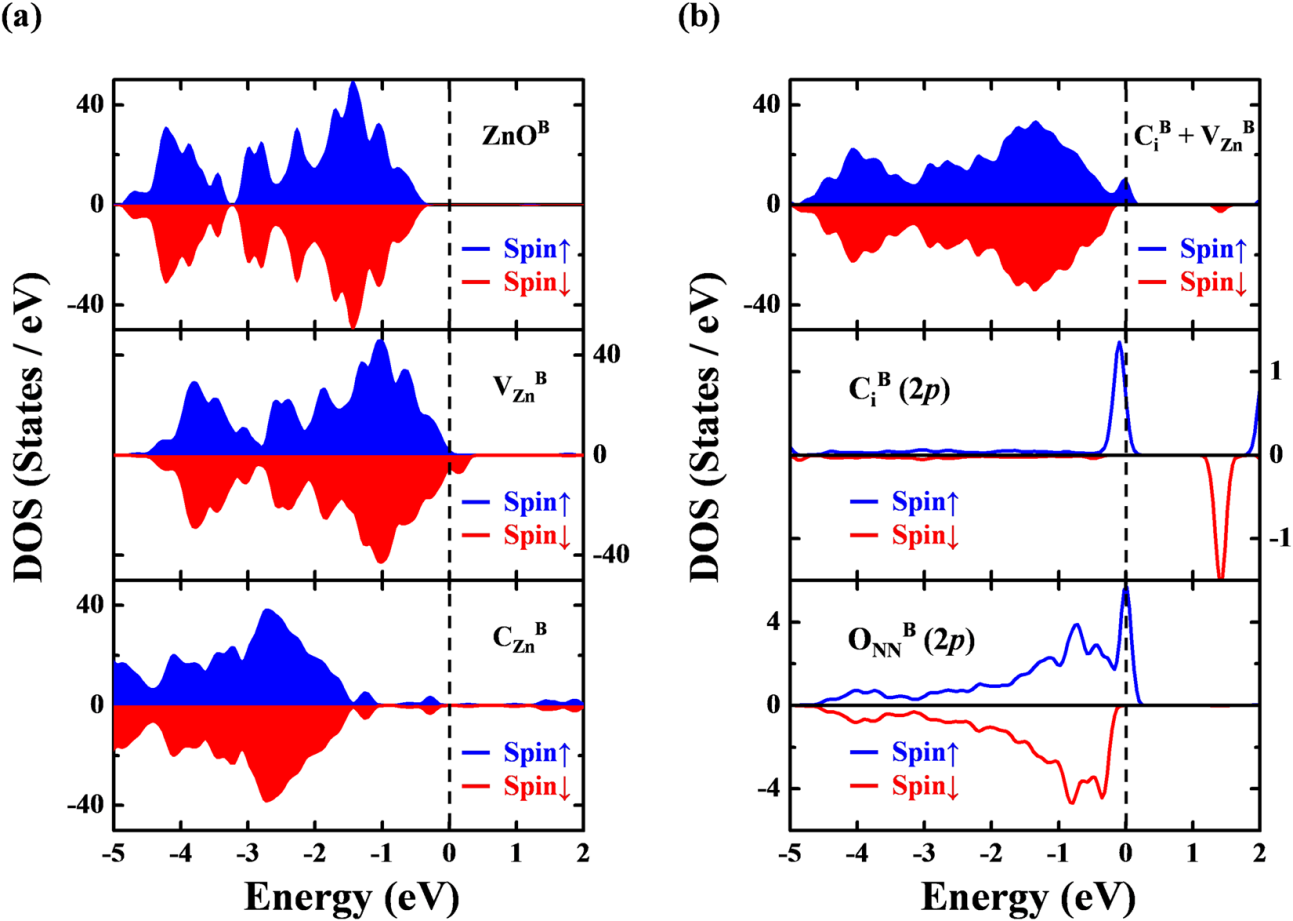
(**a**) Calculated total DOS (TDOS) of defect-free (ZnO^B^, upper panel), single Zn-vacancy (${{{\rm{V}}}_{{\rm{Zn}}}}^{{\rm{B}}}$, middle panel), and single carbon-substitution (${{{\rm{C}}}_{{\rm{Zn}}}}^{{\rm{B}}}$, lower panel) in a 72-atom supercell (Zn_36_O_36_). (**b**) Calculated TDOS (upper panel) and partial DOS (PDOS) of interstitial-vacancy complex $({{{\rm{C}}}_{{\rm{i}}}}^{{\rm{B}}}+{{{\rm{V}}}_{{\rm{Zn}}}}^{{\rm{B}}})$. 2*p* states of interstitial C atom and four nearest-neighbor O atoms are shown in middle and lower panels, respectively. Majority and minority spins are represented as blue and red areas and curves, respectively. *E*_F_ denoted as the vertical dashed line is aligned to 0 eV.

In the bulk-region (denoted by the superscript “B”) of ZnO-C:NW, a single Zn-vacancy in a 72-atom supercell was first generated by removing one Zn atom (Zn_35_O_36_). The calculated local structure around the Zn-vacancy exhibits Jahn-Teller distortion with symmetry breaking, which has been identified in other Zn chalcogenides^66^. Compared with the spin-resolved total DOS (TDOS) of vacancy-free bulk ZnO^B^ in the upper panel of Fig. 6(a), the calculated TDOS of ${{{\rm{V}}}_{{\rm{Zn}}}}^{{\rm{B}}}$, as shown in the middle panel of Fig. 6(a), reveals that two 2*p* hole states of four NN O atoms (O_NN_) around the center of Zn-vacancy induces a significant magnetic moment (~1.7 μ_B_). In the bulk environment, our calculated formation energy of ${{{\rm{V}}}_{{\rm{Zn}}}}^{{\rm{B}}}$ was 5.47 eV (1.97 eV) under Zn-rich (Zn-poor) condition, respectively, which agrees closely with that obtained elsewhere^67^. Furthermore, a single C atom that is implanted in ZnO matrix may substitute at an O site to form ${{{\rm{C}}}_{{\rm{O}}}}^{{\rm{B}}}$, occupy a Zn-vacancy site to create another substituted form, ${{{\rm{C}}}_{{\rm{Zn}}}}^{{\rm{B}}}$, or reside at an interstitial site $({{{\rm{C}}}_{{\rm{i}}}}^{{\rm{B}}})$ to form an implantation complex with a nearby Zn-vacancy, ${{{\rm{C}}}_{{\rm{i}}}}^{{\rm{B}}}+{{{\rm{V}}}_{{\rm{Zn}}}}^{{\rm{B}}}$. A previous study^68^ attributed the rareness of ${{{\rm{C}}}_{{\rm{O}}}}^{{\rm{B}}}$ defects to their high formation energy. Therefore, this work focuses on two defect configurations of C-substitution (C_Zn_^B^) and the complex ${{{\rm{C}}}_{{\rm{i}}}}^{{\rm{B}}}+{{{\rm{V}}}_{{\rm{Zn}}}}^{{\rm{B}}}$. In the case of ${{{\rm{C}}}_{{\rm{Zn}}}}^{{\rm{B}}}$, a C atom donates two electrons to annihilate two holes in ${{{\rm{V}}}_{{\rm{Zn}}}}^{{\rm{B}}}$ and thereby markedly reduces the hole-induced magnetism. This electron-hole recombination is responsible for the non-magnetic *n*-type semiconducting character of ${{{\rm{C}}}_{{\rm{Zn}}}}^{{\rm{B}}}$, as shown in the lower panel of Fig. 6(a). Also, the negative formation energy [−1.94 eV (Zn-rich) and −5.45 eV (Zn-poor)] of ${{{\rm{C}}}_{{\rm{Zn}}}}^{{\rm{B}}}$ reveals that its formation is exothermic and thermodynamically stable. However, experimental EXAFS results evidently do not support the formation of ${{{\rm{C}}}_{{\rm{Zn}}}}^{{\rm{B}}}$ upon implantation of C atoms at Zn-vacancies in ZnO:C-NW. Rather, the defect complex ${{{\rm{C}}}_{{\rm{i}}}}^{{\rm{B}}}+{{{\rm{V}}}_{{\rm{Zn}}}}^{{\rm{B}}}$ can be formed in the bulk region when the implanted C atoms reside at the interstitial sites near ${{{\rm{V}}}_{{\rm{Zn}}}}^{{\rm{B}}}$ centers, as shown in Fig. S5(a). The PDOS analysis reveals that the unbalanced DOSs in different spin channels of the defect complex ${{{\rm{C}}}_{{\rm{i}}}}^{{\rm{B}}}+{{{\rm{V}}}_{{\rm{Zn}}}}^{{\rm{B}}}$ [the upper panel of Fig. 6(b)] are attributable mainly to the antiparallel alignment of the local magnetic moment of individual ${{{\rm{C}}}_{{\rm{i}}}}^{{\rm{B}}}$ [with a net spin of 0.4 μ_B_/C_i_ attributed to its 2*p* states, as shown in the middle panel of Fig. 6(b)] and ${{{\rm{V}}}_{{\rm{Zn}}}}^{{\rm{B}}}$, which is associated with the 2*p* states of four O_NN_ [with a net spin of 0.57 μ_B_/V_Zn_, as shown in the lower panel of Fig. 6(b)]. Besides, the calculated meta-stable local structure of interstitial ${{{\rm{C}}}_{{\rm{i}}}}^{{\rm{B}}}$ is characterized by three C-O bonds (with a calculated bond length of 1.90 Å) per implanted C atom. Although the formation energy of complex ${{{\rm{C}}}_{{\rm{i}}}}^{{\rm{B}}}+{{{\rm{V}}}_{{\rm{Zn}}}}^{{\rm{B}}}$ is relatively high [4.77 eV (Zn-rich) and 1.27 eV (Zn-poor)] and its formation is clearly endothermic, such an energy barrier can be overcome by C-implantation energy (40 keV), enabling this complex to be simply generated during the C-implantation process. More interestingly, a comparison with the formation energy of a single ${{{\rm{V}}}_{{\rm{Zn}}}}^{{\rm{B}}}$ and the complex ${{{\rm{C}}}_{{\rm{i}}}}^{{\rm{B}}}+{{{\rm{V}}}_{{\rm{Zn}}}}^{{\rm{B}}}$ indicates that ${{{\rm{C}}}_{{\rm{i}}}}^{{\rm{B}}}$ can be stabilize by ${{{\rm{V}}}_{{\rm{Zn}}}}^{{\rm{B}}}$ in ZnO, with a reduction in its formation energy approximately 0.7 eV. This effect has been observed in ZnO with interstitial H atoms^67,69^.

**Figure 7:**
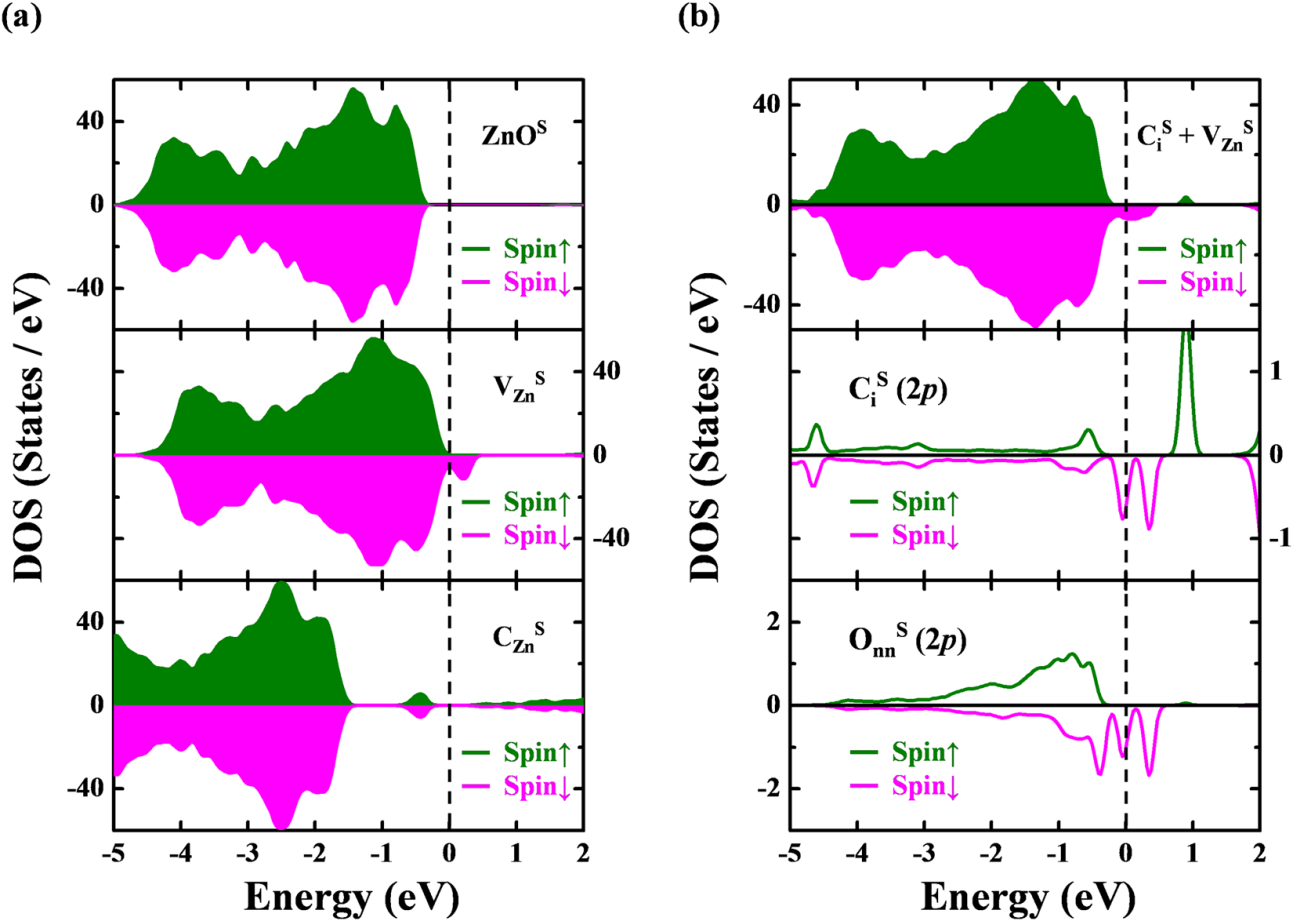
(**a**) Calculated total DOS (TDOS) of defect-free (ZnO^S^, upper panel), single Zn-vacancy (${{{\rm{V}}}_{{\rm{Zn}}}}^{{\rm{S}}}$, middle panel) and single carbon-substitution (${{{\rm{C}}}_{{\rm{Zn}}}}^{{\rm{S}}}$, lower panel) in a seven-layer atomic slab supercell (Zn_56_O_56_). (**b**) Calculated TDOS (upper panel) and partial DOS (PDOS) of vacancy-interstitial complex $({{{\rm{C}}}_{{\rm{i}}}}^{{\rm{S}}}+{{{\rm{V}}}_{{\rm{Zn}}}}^{{\rm{S}}})$. 2*p* states of interstitial C atom and four nearest-neighbor O atoms are shown in middle and lower panels, respectively. Majority and minority spins are represented as green and magenta areas and curves, respectively. *E*_F_ denoted as the vertical dashed line is aligned to 0 eV.

To model the surface-region (denoted by the superscript “S”) of ZnO-C:NW, a clean $[11\overline{2}0]$ surface of ZnO^S^ under atomic Hellmann-Feynman forces in a GGA + U scheme was optimized. A small contraction of the Zn-O bond near the surface [1.83 and 1.93 Å on the surface layer in the directions [0001] and $[1\overline{1}00]$ in Fig. S4(b), respectively] was obtained. As in the bulk-region, the implantation of a single C atom at two defect sites was considered; C-substitution at a Zn-vacancy formed ${{{\rm{C}}}_{{\rm{Zn}}}}^{{\rm{S}}}$ and a C-interstitial-vacancy complex on the surface of ZnO^S^, it formed ${{{\rm{C}}}_{{\rm{i}}}}^{{\rm{S}}}+{{{\rm{V}}}_{{\rm{Zn}}}}^{{\rm{S}}}$ [as shown in Fig. S5(b)]. As shown in the middle panel of Fig. 7(a), the holes of unpaired/dangling O atoms around the ${{{\rm{V}}}_{{\rm{Zn}}}}^{{\rm{S}}}$ center contributed to local magnetic moments (~1.65 μ_B_) on the surface of ZnO. The calculated formation energy of neutral ${{{\rm{V}}}_{{\rm{Zn}}}}^{{\rm{S}}}$ [5.24 eV (Zn-rich) and 1.73 eV (Zn-poor)] was smaller than the bulk value owing to the surface effect. C-substitution at a Zn-vacancy on the surface $({{{\rm{C}}}_{{\rm{Zn}}}}^{{\rm{S}}})$ yields a more stable defect state with a negative formation energy [−0.78 eV (Zn-rich) and −4.28 eV (Zn-poor)], which is therefore exothermic. As shown in the lower panel of Fig. 7(a), electron-hole recombination occurred in the C-substitution defects at the surface forming an *n*-type semiconductor without apparent local moment. The PDOS analysis in the case of the interstitial-vacancy complex ${{{\rm{C}}}_{{\rm{i}}}}^{{\rm{S}}}+{{{\rm{V}}}_{{\rm{Zn}}}}^{{\rm{S}}}$ at the surface, in the upper panel of Fig. 7(b), reveals opposite orientations of the local moments of individual ${{{\rm{C}}}_{{\rm{i}}}}^{{\rm{S}}}$ [with a net spin of 0.2 μ_B_/C_i_, attributed to its 2*p* state, as presented in the middle panel of Fig. 7(b)] and ${{{\rm{V}}}_{{\rm{Zn}}}}^{{\rm{S}}}$ [with a net spin of 0.9 μ_B_/V_Zn_, associated with 2*p* states of O_NN_, as shown in the lower panel of Fig. 7(b)], which significantly restrain the *d*^0^ magnetism of the ZnO-C:NW surface. Such an antiparallel magnetic moment configuration of C and O atoms is also consistent with the C and O *K*-edge XMCD measurements. Three C-O bonds with bond lengths of approximately 2.0 Å are formed by each implanted ${{{\rm{C}}}_{{\rm{i}}}}^{{\rm{S}}}$. Since the surface region contains more defects, C-implantation is expected to form more C-O bonds in the surface region than in the bulk region. This scenario agrees closely with the above arguments inference from the core-level XPS and O *K*-edge STXM-XANES spectra. Additionally, the higher formation energy of complex ${{{\rm{C}}}_{{\rm{i}}}}^{{\rm{S}}}+{{{\rm{V}}}_{{\rm{Zn}}}}^{{\rm{S}}}$ can be overcome by the high-energy of C-implantation process. As in the bulk region, the presence of C interstitials close to vacancy centers dramatically lowers the formation energy of a single ${{{\rm{V}}}_{{\rm{Zn}}}}^{{\rm{S}}}$. Importantly, the residual C-defects, especially in the bulk region, are dominated only by C_i_, based on its negligible formation energy (0.13 eV), as presented in Fig. S6 in the Supplementary Information. C_i_ not only contributes extra local moments (~0.2 μ_B_/C_i_ in C_i_-only defects in the bulk region), but also generates C-O bonds at the surface, supporting the spectroscopic results. Therefore, C_i_ still dominate the RTFM after the implantation of C atoms into ZnO-C:NW. Pan *et al*.^15^ and Bharath-Ram *et al*.^70^ investigated C-doped and C ion implanted ZnO system. They proposed that the C ions replace O site and results in Zn-C system. However, these studies do not explicitly show either C- or O-ion element specific spectroscopic techniques that C is doped at divalent O sites or vacancies. The present work demonstrates that the implanted C atoms in the ZnO-NW and ZnO-NC do not locate at O and or Zn vacancies, but remain as C interstitials.

In summary, the PL and M-H measurements revealed the modification of defects and the enhancement of magnetization in ZnO-C:NW relative to ZnO-NW. The X-ray spectroscopic studies demonstrate that (i) the interstitial C ions are the majority defects in ZnO-C:NW and interstitially bonded with O, extra C-O bonds are in the surface region, (ii) the decrease in the number of unpaired/dangling O 2*p*-derived states and thereby weakening the magnetic moments at O sites in the surface region of ZnO-C:NW compared to ZnO-NW, and (iii) the C 2*p*(π*)-derived states of the residual C(C_i_) are mainly responsible for the enhancement of RTFM in the bulk region in ZnO-C:NW compared to that in ZnO-NW. The first-principles calculations confirm that the C_i_ is stabilized by V_Zn_ in both the bulk and the surface regions of the ZnO system. However, the extra magnetic moments are provided by residual C_i_ following the C-ion implantation may have been the origin of the enhancement of RTFM in ZnO-C:NW. Finally, to consider the measurements should be also reproduced for a series of ZnO samples, the H-M curve and synchrotron-related measureemts for samples of ZnO-NC and C implanted ZnO nanocactus (ZnO-C:NC) under the same C-bombarded condition mentioned above were also carried out, further support our reuslts and conclusion stated in this work of C implanted ZnO-NW. The reproduced and consistent for ZnO-C:NC and ZnO-NC samples in comparsion to those of ZnO-C:NW and ZnO-NW were presented in Figs S7–S10 of the Supplementary Information.

## Methods

### Preparation of ZnO-NW and ZnO-C:NW

Aligned ZnO-NW were grown on seeded fluorine-doped tin oxide substrates by chemical bath deposition process using 0.02 M aqueous Zn-acetate and hexamethylenetetramine at 95 °C for three hours. The details of the synthesis, morphology and characteristics of ZnO-NW are described elsewhere^33^. C implantation was performed using the low-energy implantation facility of the Inter-University Accelerator Center, New Delhi, India. In this case, 40 keV ^12^C^+^ ions were implanted into the ZnO-NW with a fluence of 1.5 × 10^16^ ions/cm^2^. The beam current density was maintained at ~1 μA/cm^2^ throughout the implantation.

### Characterization

Field-emission SEM was performed to study the morphology of ZnO-C:NW and ZnO-NW and the effects of C implantation. Room-temperature M-H loop measurements were carried out using the superconducting quantum interference device magnetometer when a magnetic field was applied out of plane direction. The RRBS measurements were made at the Korea Institute of Science and Technology, Seoul, Korea. The ^12^C(α,α)^12^C resonant reaction with an α-particle energy of 4.27 MeV was used to measure element-depth profiles of ZnO-C:NW and ZnO-NW^46,47^. The resonant cross-section of ^12^C(α, α)^12^C reaction is approximately 120 times greater than the non-resonant cross-section^71^, and this resonant scattering can be effectively utilized to profile the depth distribution of implanted C. The backscattered α-particles were detected using a surface barrier silicon detector with a scattering angle of 170^0^ from the direction of the incident α-particles. The resonant RRBS spectra were analyzed using SIMNRA software (home.rzg.mpg.de/~mam/Manual.pdf). The atomic ratio of the elements in ZnO-C:NW and ZnO-NW was obtained from the analysis results.

The Zn *K*-edge EXAFS spectra, O *K*- and Zn *L*_3,2_-edge XANES/XMCD and core-level XPS and VB-PES spectra were obtained at the Wiggler-17C, HSGM-20A, Dragon-11A and Undulator-09A beamlines, respectively, at the National Synchrotron Radiation Research Center in Hsinchu, Taiwan. The Zn *K*-edge EXAFS spectra were obtained in fluorescence mode, while the C, O *K*- and Zn *L*_3,2_-edge XMCD spectra were obtained in surface-sensitive electron yield mode. The angle of incidence of the X-ray was fixed at 30° from the sample normal, and a magnetic field of 1 T was applied parallel and antiparallel to the sample normal throughout the XMCD measurements. The resolution was set to ~0.1 eV, 0.1 eV and 0.2 eV at photon energies of 280 eV, 530 eV and 1020 eV for the C, O *K*-edge and Zn *L*_3,2_-edge XANES measurements, respectively. The core-level XPS and VB-PES measurements were performed using a hemispherical electron-analyzer system. Photoelectrons were collected using a hemispherical analyzer with a 16-channel multichannel detector. XPS spectra were calibrated using the *E*_F_ of clean gold metal, and the energy of the incident X-ray was fixed at 380 eV for C-1*s* core-level XPS and VB-PES measurements and 630 eV for O 1 *s* core-level XPS measurements, with the energy resolution set to ~0.1 eV. The C *K*-edge XMCD spectra and the O *K*-edge STXM with corresponding XANES spectra were respectively obtained from beamline-4B and beamline-4U of the UVSOR-III Synchrotron of the Institute for Molecular Science, Okazaki, Japan. In the O *K*-edge STXM mapping measurements, the monochromatic X-ray beam was focused using a Fresnel zone plate to a ~30 nm spot onto the sample, and the sample was raster-scanned with the synchronized detection of transmission X-ray to generate a sequence of images (image stack) over the range of photon energies of interest. Energy scans of the regions of interest were conducted stepwise with a typical resolving power (E/ΔE) of ~6,000 at the O *K*-edge. The STXM data were analyzed using an aXis2000 (http://unicorn.mcmaster.ca/aXis2000.html) and PCA_GUI (http://xray1.physics.sunysb.edu/data/software.php).

## Electronic supplementary material

Supplementary Information
