# Supplementary material for: Origin of magnetic properties in carbon implanted ZnO nanowires
Source: Sci Rep. 2018 May 17;8:7758. doi: 10.1038/s41598-018-25948-x (PMC5958067; doi:10.1038/s41598-018-25948-x)
Supplement: Supplementary file 1 — Supplementary Information [file 41598_2018_25948_MOESM1_ESM.docx]

Supplementary Information

Origin of Magnetic properties in carbon implanted ZnO nanowires

Y. F. Wang^1^, Y. C. Shao^1^, S. H. Hsieh^1^, Y. K. Chang^1^, P. H. Yeh^1^, H. C. Hsueh^1,*^, J. W. Chiou^2,*^, H. T. Wang^3^, S. C. Ray^4^, H. M. Tsai^5^, C. W. Pao^5^, C. H. Chen^5^, H. J. Lin^5^, J. F. Lee^5^, C. T. Wu^6^, J. J. Wu^6^, Y. M. Chang^7^, K. Asokan^8^, K. H. Chae^9^, T. Ohigashi^10^, Y. Takagi^10^, T. Yokoyama^10^, N. Kosugi^10^, W. F. Pong^1,*^

^1^ Department of Physics, Tamkang University, Tamsui 251, Taiwan

^2^ Department of Applied Physics, National University of Kaohsiung, Kaohsiung 811, Taiwan

^3^ Department of Physics, National Tsinghua University, Hsinchu 300, Taiwan

^4^ Department of Physics, University of South Africa, Johannesburg, South Africa

^5^ National Synchrotron Radiation Research Center, Hsinchu 300, Taiwan

^6^ Department of Chemical Engineering, National Cheng Kung University, Tainan 701, Taiwan

^7^ Center for Condensed Matter Sciences, National Taiwan University, Taipei 106, Taiwan

^8^ Inter-University Accelerator Center, Aruna Asaf Ali Marg, New Delhi 110 067, India

^9^ Korea Institute of Science and Technology, Seoul 02792, Republic of Korea

^10^ Institute for Molecular Science, Okazaki 444-8585, Japan

I. Resonance Rutherford Backscattering Spectrometry (RRBS)

Figure S1(a) presents RRBS spectra that were recorded at ~4.27 MeV (α-particles) to obtain the elemental ratio depth profiles in ZnO-C:NW and ZnO-NW. Spectra indicate presence of C (~200 keV), O (~350 keV), Zn (~750 keV) and Sn (~850 keV) atoms.^1,2^ Sn signal from ZnO-C:NW arises from the substrate; concentration of Sn is less 6% so Sn has no significant effect on atomic ratio of C in ZnO-C:NW. C-depth profile [upper panel of Fig. S1(b)] reveals that C atoms in ZnO-C:NW are distributed throughout region of 0-400 nm and are concentrated at ~200 nm (indicated by red bar) below surface of sample, clearly indicating that implanted C atoms are not below surface region but also presented drastically in bulk region of ZnO nanostructure. Middle panel of Fig. S1(b) displays Zn-depth profiles in both ZnO-C:NW and ZnO-NW, which do not show significant differences in atomic distribution of Zn, suggesting that implanted C atoms do not significantly alter distribution or dislocation of Zn atoms. Depth profile of O atoms in lower panel of Fig. S1(b) is not useful for analysis owing to low scattering cross-section of O atoms in RRBS spectra that were obtained at ~4.27 MeV.

Fig. S1 (a) RRBS spectra and (b) depth profiles of ZnO-C:NW and ZnO-NW.

II. Phase and phase derivative analysis of Zn *K*-edge EXAFS spectra

Figures S2(a) and S2(b) display the phase and phase derivative analysis^3-5^ to verify quantitatively whether the implanted C atoms replace vacancies at either V_O_ and V_Zn_ sites in ZnO nanostructures. Phase derivative analysis has been performed extensively on distorted perovskites,^4^ and provides accurate information about atoms that are present at various sites with slightly different bond distances, yielding a *beating point* (k_b_) in the EXAFS oscillations. The wavenumber at which beating occurs (in phase derivative analysis) is given by

$\mathbf{k}_{\mathbf{b}}\boldsymbol{\sim}\frac{\boldsymbol{\pi}}{\boldsymbol{2\Delta R}}$ $\mathbf{k}_{\mathbf{b}}\boldsymbol{\sim}\frac{\boldsymbol{\pi}}{\mathbf{2}\boldsymbol{\Delta}\mathbf{R}}$

in the first and second shells, where ∆R is the difference between the two bond distances at different sites.

Accordingly, if the implanted C atoms truly substituted the vacancies at either V_O_ and V_Zn_ sites to form Zn-C bonds in corresponding first and second shells, then the beating point k_b_ would be observed and extractable from the FT spectra of NN Zn-O and Zn-Zn in Fig. 2(a). However, the inverse FT spectra [Figs. S2(a)-(d)] and phase derived analysis [Figs. S2(e)-(h)] of first shell Zn-O and second shell Zn-Zn bond lengths in surface and bulk regions of ZnO-C:NW and ZnO-NW are almost identical of extracted phase function Ψ(*k*) and phase derivative function dΨ/d*k*, no beating point is observed in EXAFS oscillations of ZnO-C:NW, thus excluding possibility that implanted C atoms substitute at V_O_ and V_Zn_ sites to form Zn-C bonds in either first or second shells of ZnO-C:NW. This finding confirms the presence of interstitial defects of C_i_ in the ZnO-C:NW nanostructures upon implantation of C atoms.

Fig. S2(a)-(d) Inverse Fourier transform (FT) spectra in Fig. 2(a) and (e)-(h) phase derived analysis of nearest-neighbor (NN) Zn-O (bond length ~1.8 Å) and NN Zn-Zn bonds (bond length ~3.2 Å) in surface (θ= 80^o^) and bulk (θ = 0^o^) regions of ZnO-C:NW and ZnO-NW.

III. Valence-band photoemission spectroscopy

Figure S3 displays valence-band photoemission (VB-PES) spectra at/below valence-band maximum (*E*_VBM_) or Fermi level (*E*_F_) of ZnO-C:NW (red) and ZnO-NW (blue), which were obtained using an incident photon energy of 380 eV. Zero energy is chosen to be at the *E*_VBM_ or *E*_F_, which is the threshold of the emission spectrum. General line-shapes of VB-PES spectra of ZnO-C:NW and ZnO-NW are similar to those that have been obtained previously from aligned ZnO nanorods.^6,7^ Clearly, Fig. S3 reveals that intensities with binding energy of around 3-6 eV in VB-PES spectra of ZnO-C:NW are considerably lower than those in VB-PES spectra of ZnO-NW. Since occupied states at/below *E*_VBM_ or *E*_F_ are dominated by defects and dangling/unpaired bonds of anion-derived *p* states, which are O 2*p*-derived states in ZnO nanostructures,^8^ reduction of VB-PES intensities around 3-6 eV in ZnO-C:NW can be attributed to reduction of dangling/unpaired O 2*p*-derived states herein. In the present case implanted C atoms bonded with O around intrinsic defect sites in the surface region of ZnO-C:NW. This could be the effect for reducing the density of O 2*p*-derived states at near/below the *E*_VBM_ or *E*_F_ of ZnO-C:NW compare to that of ZnO-NW, as shown in VB-PES spectra.

Fig. S3 VB-PES spectra at/below valence-band maximum (*E*_VBM_) or Fermi level (*E*_F_) of ZnO-C:NW (red) and ZnO-NW (blue).

IV. Computational details

The ground-state electronic and magnetic structures of various neutral defects in bulk and surface regions of wurtzite ZnO were calculated by the first-principles projector augmented wave (PAW) method,^9^ using the Vienna *ab initio* simulation package (VASP).^10^ These calculations are based on spin-polarized density-functional theory (DFT) with a generalized-gradient approximation (GGA) and the Perdew-Burke-Ernzerh (PBE) functional.^11^ The plane-wave energy cutoff was set to 750 eV. The experimental lattice parameters (***a***=***b***= 3.249 **Å**, ***c***= 5.205 **Å**)^12^ of the primitive wurtzite cell of ZnO are used to enable direct comparison with observations. To deal with the insufficiency of description of the strongly correlated *d*-orbital of Zn in the mean-field LDA/GGA approximation, which overestimates *pd* hybridization and significantly underestimates the band gap in bulk ZnO, a rotationally invariant spin-polarized GGA+U method^13^ with Coulomb screening and exchange parameters (*U-J* = 7.0 eV) was adopted. The underestimated GGA band gap (0.75 eV) of bulk wurtzite ZnO is improved by the GGA+U calculation which calculated band-gap value of 1.76 eV is closer to the experimental value (3.6 eV).^14^ To model the various defect configurations in ZnO-C:NW following the implantation of C atoms in both bulk and surface regions, the supercell scheme that is composed of periodic repetitions of the primitive wurtzite ZnO unit cell is used. Taking into account the finite-size effect^15^ and the effect of incomplete error cancellation^16^ on the total energy of the supercells, the formation energy of large supercells was calculated with corrections:^16^ a 72-atom 3×3×2 unit cell of wurtzite ZnO [as shown in Fig. S4(a)] is used in the bulk region whereas a 2×2 periodic slab that is composed of 112 atoms [seven atomic layers with a vacuum region of 15 **Å,** as shown in Fig. S4(b)] is constructed to model the ZnO $\left[ 11\bar{2}0 \right]$ $\left[ 11\bar{2}0 \right]$ $\left[ 11\bar{2}0 \right]$ surface. Structural relaxations of bulk and surface systems with neutral defects were allowed until interatomic forces reached less than 0.002 eV/**Å**. In particular, to avoid the identification a fictitious electrostatic interaction between adjacent replicas,^17^ point defects were arranged symmetrically on both sides of the slab and the outer two layers were allowed to relax with the central three layers anchored to their bulk positions. Monkhorst-Pack *k*-grid sampling^18^ with 2×2×4 and 5×5×1 grids was carried out to obtain Brillouin zone integrals for calculating bulk and surface supercell calculations, respectively, to ensure convergence.

**Formation energy calculations**

The formation energy (*E ^f^* $\mathbf{E}^{\mathbf{f}}$) of point defects *D* in the neutral state of ZnO is determined by

*E ^f^* (*D*) = *E_tot_* (*D; ZnO*) － *E_tot_* (*ZnO*) + Σ*_D_ n_D_ μ_D_*

where *E_tot_* (*D; ZnO*) is the total energy of the ZnO supercell that contains defect *D* in the neutral state; *E_tot_* (*ZnO*) is the total energy of a perfect ZnO crystal in the same supercell; *n_D_* is the number of point defects *D* that are removed (positive) or added (negative) during defect formation, and *μ_D_* is the chemical potential of element *D*, which is related to the experimental growth conditions. The chemical potential of Zn (*μ_Zn_*) and O (*μ_O_*) atoms under thermodynamic equilibrium must meet the criterion

Δ*H_f_* (*ZnO*) = *μ_Zn_* + *μ_O_*

where **Δ*H_f_* (*ZnO*)** is the enthalpy of formation of bulk ZnO. The upper bounds of ***μ_Zn_*** and ***μ_O_*** can be obtained from the total energies of the bulk *hcp*-Zn [***E_tot_* (*Zn_hcp_*)**] and the O molecule [***E_tot_* (*O_2_*)**], respectively. Including the optimized error cancellation in the GGA+U total energies,^16^ our calculated formation enthalpy of ZnO (-3.504 eV) is in good agreement with the experimental value of -3.631 eV.^19^ To reflect the experimental conditions herein, ***μ_Zn_*** of the Zn vacancy ranges from that for the Zn-rich condition [***μ^max^_Zn_*** = ***E_tot_* (*Zn_hcp_*)**] to that for the Zn-poor condition [$\mu_{\mathrm{Zn}}^{\min}=E_{\mathrm{tot}}\left( \mathrm{Zn}_{\mathrm{hcp}} \right)+\Delta H_{f}\left( \mathrm{ZnO} \right)$***μ^min^_Zn_*** = ***E_tot_* (*Zn_hcp_*) + Δ*H_f_* (*ZnO*)**] in both bulk and surface regions. A limiting chemical potential of the C-rich condition [***μ^max^_C_*** = ***E^atomic^_tot_* (*C*)**] is adopted to specify the condition of carbon atom implantation.

To visualize the effects of defects on both electronic and local structures, Figs. S5(a) and (b) show the spin-density distribution of an interstitial-vacancy complex (C_i_+V_Zn_) in the bulk and the surface regions, respectively. The net spin density with spin-up/spin-down in Figs. S5(a) and (b) is represented using yellow/cyan isosurfaces near the center of defect, and Zn, O, and C atoms are illustrated as large-grey, small-red, and middle-blue spheres, respectively. Figure S6 presents the formation energies of Zn vacancies (V_Zn_), the interstitial-vacancy complex (C_i_+V_Zn_), and C-substitution (C_Zn_) at various regions (bulk/surface regions outlined in solid/dashed lines, respectively) under both Zn-rich and Zn-poor growth conditions. Clearly, the C interstitials (whose formation in the bulk region is denoted as a green dot-dashed line) further reduced the formation energy of Zn vacancies and so stabilizes them. The lower formation energies of both V_Zn_ and C_i_+V_Zn_ in the surface region than in the bulk region imply a higher defect concentration in the former. This finding is consistent with RRBS spectra in Fig. S1(b). However, a large population of C ions that is generated by implantation could easily occupy the Zn vacancies to form C_Zn_ defects, owing to their significantly low formation energies in both bulk and surface regions, as indicated by the blue solid/dashed lines in Fig. S6. Finally, the residual C defects, especially in the bulk region, are dominated by C interstitials (C_i_), owing to their negligible formation energy (0.13 eV).

**(a) (b)**

**Fig. S4** Illustration of wurtzite ZnO crystal structure in the configuration of (a) bulk and (b) surface with a $\left[ 11\bar{2}0 \right]$ surface orientation. Zn and O atoms are represented as large-grey and small-red, respectively.

**(a)** **(b)**


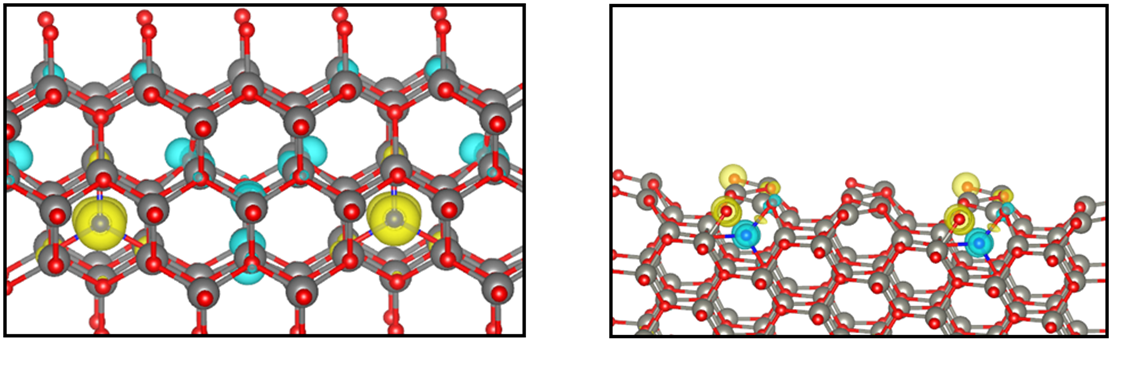


**Fig. S5** The spin density of vacancy-interstitial complex (C_i_ +V_Zn_) in (a) bulk and (b) surface regions. The net spin-up/spin-down density is indicated by yellow/cyan isosurfaces near defect centers. Zn, O and C atoms are depicted as large-grey, small-red, and middle-blue spheres, respectively.

**Fig. S6** (color online) Calculated formation energy of vacancy (V_Zn_), interstitial-vacancy complex (C_i_+ V_Zn_), C-substitution (C_Zn_) in the bulk (solid lines) and surface (dashed lines) at different experimental growth conditions (Zn-rich and Zn-poor). The formation of C interstitial (C_i_) defect in bulk region is denoted as a green dot-dashed line for reference.

References

1. Murmu, P. P. *et al*. Effect of annealing on the structural, electrical and magnetic properties of Gd-implanted ZnO thin films. *J*. *Mater*. *Sci*. 47, 1119-1126 (2012).
2. Jung, K. *et al*. Highly conductive and damp heat stable transparent ZnO based thin films for flexible electronics. *J*. *Alloys Compd*. 554, 240-245 (2013).
3. Martens, G., Rabe, P., Schwentner, N. & Werner, A. Extended X-ray-absorption fine-structure beats: A new method to determine differences in bond lengths. *Phys*. *Rev*. *Lett*. 39, 1411-1414 (1977).
4. Piamonteze, C. *et al*. Short-range charge order in RNiO_3_ perovskites (R = Pr, Nd, Eu, Y) probed by X-ray-absorption spectroscopy. *Phys*. *Rev*. *B* 71, 012104 (2005).
5. Wang, B. Y. *et al*. Effect of geometry on the magnetic properties of CoFe_2_O_4_–PbTiO_3_ multiferroic composites. *RSC Adv*. 3, 7884-7893 (2013).
6. Chiou, J. W. *et al*. Electronic structure of ZnO nanorods studied by angle-dependent X-ray absorption spectroscopy and scanning photoelectron microscopy. *Appl*. *Phys*. *Lett*. 84, 3462-3464 (2004).
7. Chiou, J. W. *et al*. Diameter dependence of the electronic structure of ZnO nanorods determined by X-ray absorption spectroscopy and scanning photoelectron microscopy. *Appl*. *Phys*. *Lett*. 85, 3220-3222 (2004).
8. Singh, S. B. *et al*. Observation of the origin of d^0^ magnetism in ZnO nanostructures using X-ray-based microscopic and spectroscopic techniques. *Nanoscale* 6, 9166-9176 (2014).
9. Blöchl, P. E. Projector augmented-wave method. *Phys. Rev. B* 50, 17953-17979 (1994).
10. Kresse, G. & Hafner, J. Ab initio molecular dynamics for liquid metals. *Phys. Rev. B* 47, 558-561 (1993).
11. Perdew, J. P., Burke, K. & Ernzerhof, M. Generalized Gradient Approximation Made Simple. *Phys. Rev. Lett.* 77, 3865-3868 (1996).
12. Schulz, H. & Thiemann, K. H. Crystal structure refinement of AlN and GaN. *Solid State Commun.* 23, 815-819 (1977).
13. Dudarev, S. L., Botton, G. A., Savrasov, S. Y., Humphreys, C. J., & Sutton, A. P. Electron-energy-loss spectra and the structural stability of nickel oxide: An LSDA+U study. *Phys. Rev. B* 57, 1505-1509 (1998).
14. Shih, B. C., Xue, Y., Zhang, P., Cohen, M. L. & Louie, S. G. Quasiparticle band gap of ZnO: High accuracy from the conventional G^0^W^0^ approach. *Phys. Rev. Lett.* 105, 146401 (2010).
15. Erhart, P., Albe, K. & Klein, A. First-principles study of intrinsic point defects in ZnO: Role of band structure, volume relaxation, and finite-size effects. *Phys. Rev. B* 73, 205203 (2006).
16. Stevanović, V., Lany, S., Zhang, X. & Zunger, A. Correcting density functional theory for accurate predictions of compound enthalpies of formation: Fitted elemental-phase reference energies. *Phys. Rev. B* 85, 115104 (2012)
17. Calzolari, A. & Catellani, A. Water adsorption on nonpolar ZnO [$\boldsymbol{10}\bar{\boldsymbol{1}}\boldsymbol{0}$] surface: A microscopic understanding. *J. Phys. Chem. C* 113, 2896-2902 (2009)
18. Monkhorst, H. J. & Pack, J. D. Special points for Brillouin-zone integrations. *Phys. Rev. B* 13, 5188-5192 (1976).
19. Dean, A. J. *Lange’s Handbook of Chemistry*. 15th edn., McGraw-Hill, New York (1999).
